# Intensification of 2′-Fucosyllactose biosynthesis pathway by using a novel fucosyltransferase from *Bacillus cereus*

**DOI:** 10.3389/fbioe.2025.1569597

**Published:** 2025-04-30

**Authors:** Kainuo Zhang, Miaomiao Gao, Chenqi Cao, Mengxin Zhang, Waqar Ahmad, Ahmed Rady, Badr Aldahmash, Tianze Zhu, Shahin Shah Khan, Luo Liu

**Affiliations:** ^1^ College of Life Science and Technology, Beijing University of Chemical Technology, Beijing, China; ^2^ Department of Zoology, College of Science, King Saud University, Riyadh, Saudi Arabia; ^3^ Beijing Zeno Biotechnology Development Co. Ltd., Beijing, China

**Keywords:** 2′-fucosyllactose, *de novo* synthesis, human milk oligosaccharide, fermentation, FutCB

## Abstract

**Introduction:**

2′-Fucosyllactose (2′-FL) is an oligosaccharide that can be synthesized in the human body and is known for its health-promoting and prebiotic effects. The biosynthesis of 2′-FL using microorganisms has received attention recently due to its increased application in nutritional and medical infant formulations.

**Methods:**

This work attempts the new application of *Bacillus cereus* α-1,2-fucosyltransferase (FutCB) in the *de novo* synthesis of 2′-FL in *Escherichia coli* (*E. coli*). Additionally, knocking out the *LacZ* and *WaaF* genes alongside overexpression of the key gmd, manB, wcaG, and manC genes enhances the availability of the necessary precursors GDP-L-fucose and lactose for the synthesis of 2′-FL.

**Results and discussion:**

The use of constitutive promoters achieved better control over the production of 2′-FL during fed-batch fermentation. After 64 h of fermentation, the modified *E. coli* strains produced 121.4 g/L 2′-FL with a yield of 1.90 g/L/h, resulting in an impressive 2′-FL output. These results together indicate the potential of large-scale, high-yield production of 2′-FL and form a basis of much more refinement to be done. The next step will focus on maximum substrate utilization, alteration of gene regulation, and improvement of commercial-scale synthesis.

## Highlights

• *Escherichia coli* based microbial cell factory was used for *de novo* biosynthesis of 2′-FL from *B. cereus* by α-1,2-fucosyltransferase (FutCB).
• By knocking out *lacZ* and *waaF* along with overexpressing manB, manC, gmd, and wcaG, lactose and GDP-L-fucose were accumulated to enhance 2′-fucosyllactose (2′-FL) biosynthesis.
• The incorporation of a constitutive promoter significantly enhanced the stability of 2′-FL biosynthesis during fed-batch cultivation.
• The fed-batch cultivation achieved a peak 2′-FL production of 121.4 g/L after 64 h, with a production rate of 1.90 g/L/h.

## 1 Introduction

Human milk oligosaccharides (HMOs) are natural bioactive components found in breast milk. Lately, they’ve been getting a lot of attention for their potential uses in food additives and medicine (Wan et al., 2021; Zhang et al., 2022; Zhou et al., 2022; Zhang et al., 2023). 2′-FL is the most common HMOs, making up about 30% of all HMOs and is considered as the third most abundant solid in breast milk. It’s incredibly important for the growth and development of babies, helping to balance their gut microbiota, fight off harmful bacteria, support healthy gut function, and boost both their immune system and brain development (Vandenplas et al., 2018). It has received approval as a food additive for infant products from regulatory authorities, including the U.S. Food and Drug Administration (FDA), the Novel Foods and Food Allergens (NDA) panel, and China’s National Health Commission (EFSA Panel on Nutrition et al., 2019; EFSA Panel on NutritionN.F. et al., 2022; Liu et al., 2022). Relevant clinical studies have demonstrated its safety and human tolerance when used in formula preparations (SHI and JIANG, 2020). The use of 2′-FL in dietary supplements, infant formula and medical food rise the need for its scalable production. Various approaches are available for the production of 2′-FL synthesis, such as chemical synthesis (Agoston et al., 2019), enzymatic processes (Albermann et al., 2001), and precision fermentation. Among these, biosynthesis is cost effective and also suitable for industrial production is considered as the preferred method for 2′-FL synthesis.

A variety of microorganisms deemed Generally Recognized as Safe (GRAS) have been applied in the production of 2′-FL. Researchers introduced a bi-functional enzyme (*fkp*) in *S. cerevisiae* (*Saccharomyces cerevisiae*) from *Bacteroides fragilis* having GDP-L-fucose phosphorlase/L-fucokinase activity, along with lactate permease (lac12) from *Kluyveromyces lactis*. This setup enables the whole-cell production of 2′-FL via salvage pathway, successfully achieving a total yield of 503 mg/L of 2′-FL for the first time by using *S. cerevisiae* (Yu et al., 2018). 2′-FL production was also achieved via biosynthetic pathways in *P. pastoris* and *S. cerevisiae* (Hollands et al., 2019). A dual-functional gene regulation circuit was developed to control expression at transcriptional/translational levels (Deng et al., 2019b). When applied to 2′-FL biosynthesis in *Bacillus subtilis*, this system significantly increased production from 24.7 mg/L to 674 mg/L and further to 5.01 g/L (Deng et al., 2019a). Researchers achieved 2′-FL yields of 0.28 g/L, 28.6 g/L, and 27.53 g/L in *Pichia pastoris*, *Priestia megaterium*, and *Escherichia coli* BL21 (DE3), respectively (Qian et al., 2023; Park et al., 2025; Zhu et al., 2025).

2′-FL is produced in microbial hosts either by *de novo* or salvage pathway. However, the *de novo* synthesis is cost-effective (Shi et al., 2021). In *E. coli*, GDP-L-fucose, one of the essential precursors, is naturally produced from glucose or glycerol. This process involves a series of enzymes, including glucose-6-phosphate isomerase (Pgi), mannose-6-phosphate isomerase (manA), phosphomannose mutase (manB), mannose-1-phosphate guanylyltransferase (manC), GDP-mannose-4,6-dehydratase (gmd), and NADPH-dependent GDP-L-fucose synthase (wcaG) (Albermann et al., 2000; Liu et al., 2022). It is also an important precursor for the production of colonic acid (CA), an important cell envelope component of *E. coli* contributing to the survival pathogenesis and colonization of bacteria (Qiao et al., 2021). Since CA and 2′-FL share the same precursor, methods that boost CA production can also help increase 2′-FL yields. Once GDP-L-fucose is produced and lactose is taken up, the final step involves α-1,2-fucosyltransferase enzyme. However, this enzyme is often considered the bottleneck in the process (Li et al., 2020; Wan et al., 2021; Lee et al., 2022). Researchers are actively working to find more effective and efficient way for the production of α-1,2-fucosyltransferase with improved solubility (Zeuner and Meyer, 2020; Lin et al., 2022; Park et al., 2022). The biosynthesis of 2′-FL necessitates three essential components: the donor substrate GDP-L-fucose, the acceptor substrate lactose, and the enzyme α-1,2-fucosyltransferase. *E*. *coli* synthesizes GDP-L-fucose *de novo* from 6-phosphofructose via a five-step enzymatic pathway that includes the enzymes manA, manB, manC, gmd, and wcaG (Ni et al., 2020). The *de novo* synthesis pathway uses glycerol, a cheap carbon source, to produce the key precursor GDP-L-fucose, making it a cost-effective option for industrial production. To prevent the production strains from breaking down lactose, a strategy is used to deactivate β-galactosidase while also improving the strains’ ability to take up lactose (Baumgärtner et al., 2013; Sun et al., 2023).

In this study, we built on previous research to explore how gene knockouts affect the growth rate of *E. coli* C43 (DE3). To achieve the highest growth rate, this study focused on knocking out just two genes: β-galactosidase (*lacZ*) and ADP-heptose-LPS heptosyltransferase II (*waaF*). Further, boosted the GDP-L-fucose synthesis pathway by overexpressing several key enzyme genes manA, manB, gmd, wcaG, zwf (glucose-6-phosphate dehydrogenase), *fkp*, and RcsB by replacing their constitutive promoters. To further improve efficiency, we used FutCB from *B. subtilis* to create a high-performance cell factory for producing 2′-FL.

## 2 Materials and methods

### 2.1 Bacterial strains and chemical reagents

The primary *E. coli* strain C43 (DE3) was subsequently engineered to improve the biosynthesis of 2′-FL. Initially, the activation and plate screening of the strains were performed with LB medium. While Strains were thoroughly engineered utilizing CRISPR/Cas9 (Inovogen, Beijing) and subsequently cultivated in Terrific Broth (Yuanye, Shanghai) for growth and batch fermentation experiments. The expression of λ-red recombinase was achieved through the pCas plasmid (Inovogen, Beijing), induced with 40 mM L-arabinose to enable the integration of homologous fragments. To remove the sgRNA plasmid pTarget, the culture was incubated overnight in LB medium containing 10 mM rhamnose. The selection of positive strains and plasmid replication was performed while maintaining the appropriate antibiotics (100 μg/ml ampicillin, 50 μg/ml kanamycin) in the growth medium. 2′-FL was obtained from Carbosynth, a seamless cloning kit from Beyotime, and additional reagents, including cAMP, from Sangon Biotech.

### 2.2 Plasmid’s construction

The plasmids, strains, and primers utilized in this study are outlined in Table 1; Supplementary Tables S1, S2, respectively. The *E. coli* C43 (DE3) strain was utilized as the host, with pRSFDuet-1 and pETDuet-1 used for cloning and subcloning purposes. The expression of the FutC gene was regulated by either the T7 or Ptac promoter located upstream of TrxA in the pET plasmid. The pET-tac_TrxA_FutC/FutCB plasmid was constructed by integrating a Ptac promoter-TrxA-FutC gene expression cassette into the pET vector via a Seamless Cloning Kit. A fusion protein comprising TrxA at the N-terminus of FutC was engineered to improve FutC expression and facilitate the production of 2′-FL. The TrxA sequence, amplified from *E. coli* MG1655 with TrxA-FutC-F/R primers, was cloned into pET-tac-FutC, resulting in the construct pET-tac_TrxA_FutC (refer to supplementary materials). Colony PCR and DNA sequencing validated the successful construction of the strain and plasmid (Chen et al., 2013).

**TABLE 1:** Plasmids used in this study.

Plasmids	Descriptions	References
pRSFDuet-1	Kan^R^, two T7 promoter, two *lac* operator	Genscript
pRSF-CBGW	pRSFDuet-1, T7 promoter-1 ligated with manB, manC, and T7 promoter-2 ligated with gmd, wcaG gene	This study
pETDuet-1	Amp^R^, two T7 promoter, two *lac* operator	Genscript
pET-T7FutCB	pETDuet-1, T7 promoter-2 ligated with FutCB gene	This study
pET-T7FutC	pETDuet-1, T7 promoter-2 ligated with FutC gene	This study
pET-tac_TrxA_FutCB	pETDuet-1 with tac promoter linked with TrxA_FutCB gene	This study
pET-tac_TrxA_FutC	pETDuet-1 with tac promoter linked with TrxA_FutC gene	This study
pET-T7*lacY*-T7FutCB	pET-T7FutCB, T7 promoter-1 ligated with *lacY* gene	This study
pET-T7*lacY*-T7FutC	pET-T7FutC, T7 promoter-1 ligated with *lacY* gene	This study
pET-T7*lacY*-tac_TrxA_FutCB	pET-tac_TrxA_FutCB, T7 promoter-1 ligated with *lacY* gene	This study
pET-T7*lacY*-tac_TrxA_FutC	pET-tac_TrxA_FutC, T7 promoter-1 ligated with *lacY* gene	This study
pACYCDuet-1	Cm^R^, two T7 promoter, two *lac* operator	Genscript
pET-BCGW-*zwf*	pETDuet-1, J23118 promoter ligated with manB (RBS), manC (RBS: BBa-B0030), and J23104 promoter ligated with gmd (RBS: BBa-B0032), wcaG (RBS: BBa-B0064) gene	This study
pAC-FFGR-*lacY*	pACYCDuet-1, J23111 promoter ligated with FutCB (RBS), *fkp* (RBS: BBa-B0032), and J23107 promoter ligated with *gsk* (RBS: BBa-B0032), RcsB gene	This study

### 2.3 Gene knock-in/knock-out

The construction of knock-in/knock-out strains were generated with CRISPR/Cas9 as previously described (Jiang et al., 2015). The primer sequences from the template plasmid pTargetF were designed (Supplementary Tables S2) that targeted the specific integration and deletion loci for the sgRNA plasmid.

The FutC was integrated into *E. coli* C43 (DE3), by obtaining the upstream/downstream homologous arms from the *E. coli* C43 (DE3) genome. The Ptac-FutC gene was then amplified using specific primers. The necessary homologous fragments for the integration of *fkp*, TrxA-FutC and FutC genes were assembled with fusion PCR for both the knock-in/knock-out genes. The yjiP locus is a known stable integration site in *E. coli* (Goormans et al., 2020) was used for the integration of TrxA-FutC gene cassette. To knockout the *LacZ*, *wcaJ*, *wcaZ*, *wcaI*, and *waaF* genes, the same protocol was used for the assembly of homologous arms to knock-out *lacZ*, *wcaJ*, *wcaZ*, *wcaI* and *waaF* genes.

### 2.4 Fed-batch fermentation

*Escherichia coli* C43 (DE3) was cultivated in a 3-L fermenter containing 1.5 L of medium. The medium composition was as follows: glycerol (10 g/L), K_2_HPO_4_·3H_2_O (7.5 g/L), yeast extract (7.5 g/L), citric acid (2.0 g/L), MgSO_4_·7H_2_O (2.0 g/L), (NH_4_)_2_SO_4_ (1.6 g/L), and a trace element solution (10 mL/L). The trace element solution consisted of Na_2_B_4_O_7_·10H_2_O (0.23 g/L), CoCl_2_·6H_2_O (0.4 g/L), CuSO_4_·5H_2_O (1.0 g/L), ZnSO_4_·7H_2_O (2.25 g/L), and FeSO_4_·7H_2_O (8 g/L). Cultivation parameters were 30°C and pH 6.8, maintained by addition of 50% (w/v) NH_4_OH.

### 2.5 Analytical method

Plasmids utilized in this study engineered *E. coli* strain was grown in 20 mL of LB medium containing 10 g/L fucose and 4 g/L lactose. The growth conditions included a temperature of 37°C, shaking at 220 rpm, and a duration of 72 h. Cell growth was assessed using OD600 measurements. A 1 mL sample was collected for 2′-FL analysis after 72 h. The production of 2′-FL in the plasmid-free *E. coli* C43 (DE3) strain was quantified using LC-MS analysis of the fermentation supernatant. 2′-FL was identified through the comparison of mass spectrometry data with a standard, and its concentration was quantified using a standard curve derived from peak area analysis. Extracellular metabolites, including lactose, fucose, 2′-FL, glycerol, acetic acid, and mannose, were quantified via HPLC utilizing an Agilent 1260 system. The system was equipped with a Rezex™ ROA-Organic Acid H+ (8%) column and a refractive index detector. The HPLC conditions included a mobile phase of 5 mM H_2_SO_4_, a flow rate of 0.6 mL/min, and a column temperature of 55°C. All experiments were performed in triplicate.

## 3 Results and discussion

### 3.1 Construction of 2′-FL synthesis strains

**FIGURE 1:**
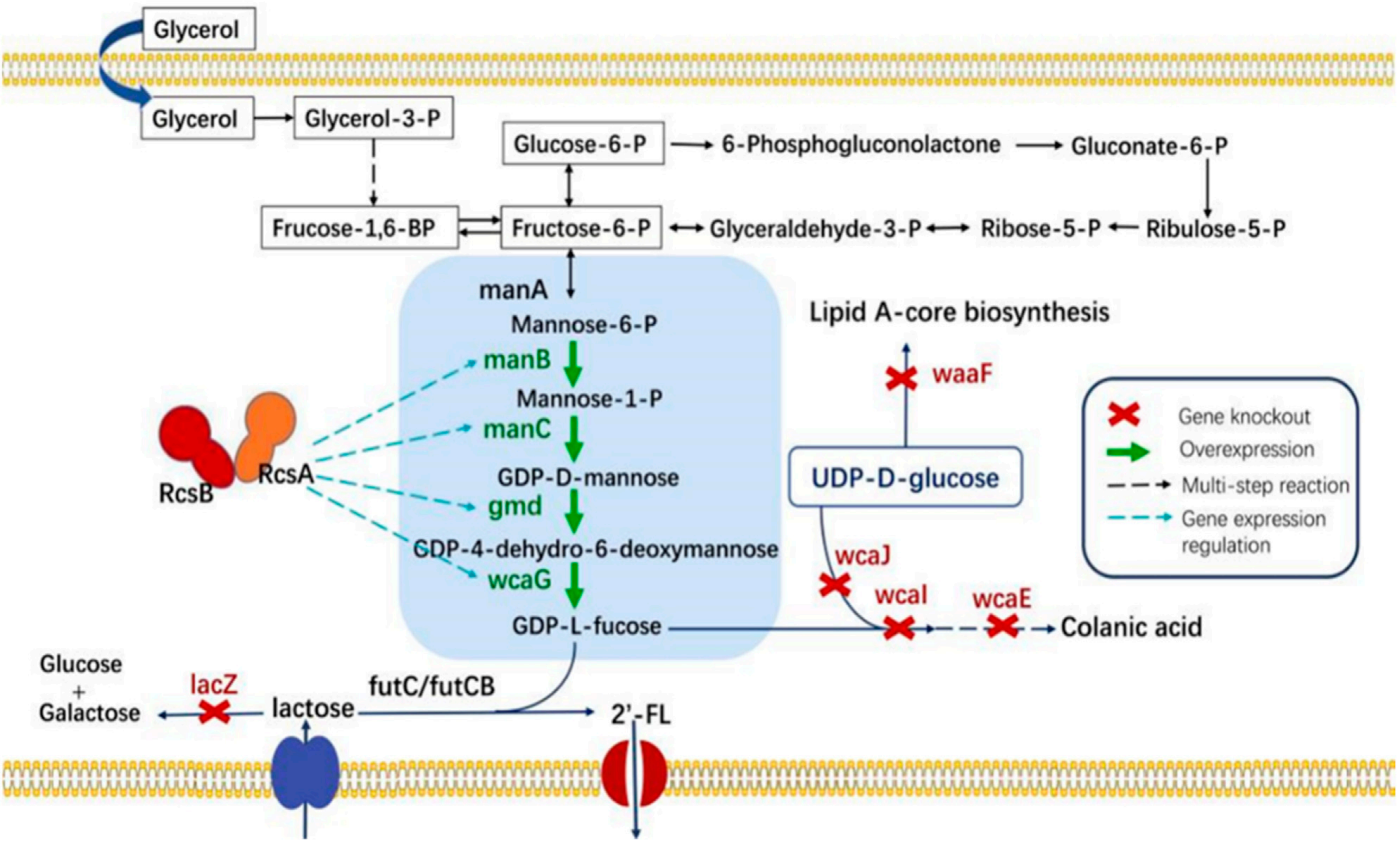
The *de novo* biosynthetic pathway for 2′-FL utilizes glycerol as the sole carbon source. Key gene abbreviations include: manA, encoding mannose-6-phosphate isomerase; manB, encoding phosphomannomutase; manC, encoding mannose-1-phosphate guanyltransferase; gmd, encoding GDP-mannose-4,6-dehydratase; wcaG, encoding NADPH-dependent GDP-L-fucose synthase; RcsA and RcsB, positive transcriptional regulators A and B; *lacZ*, encoding β-galactosidase; *wcaJ*, encoding UDP-glucose lipid carrier transferase; *wcaI* and *wcaE*, involved in colanic acid biosynthesis; and *waaF*-ADP-heptose, the LPS heptosyltransferase II.

**FIGURE 2:**
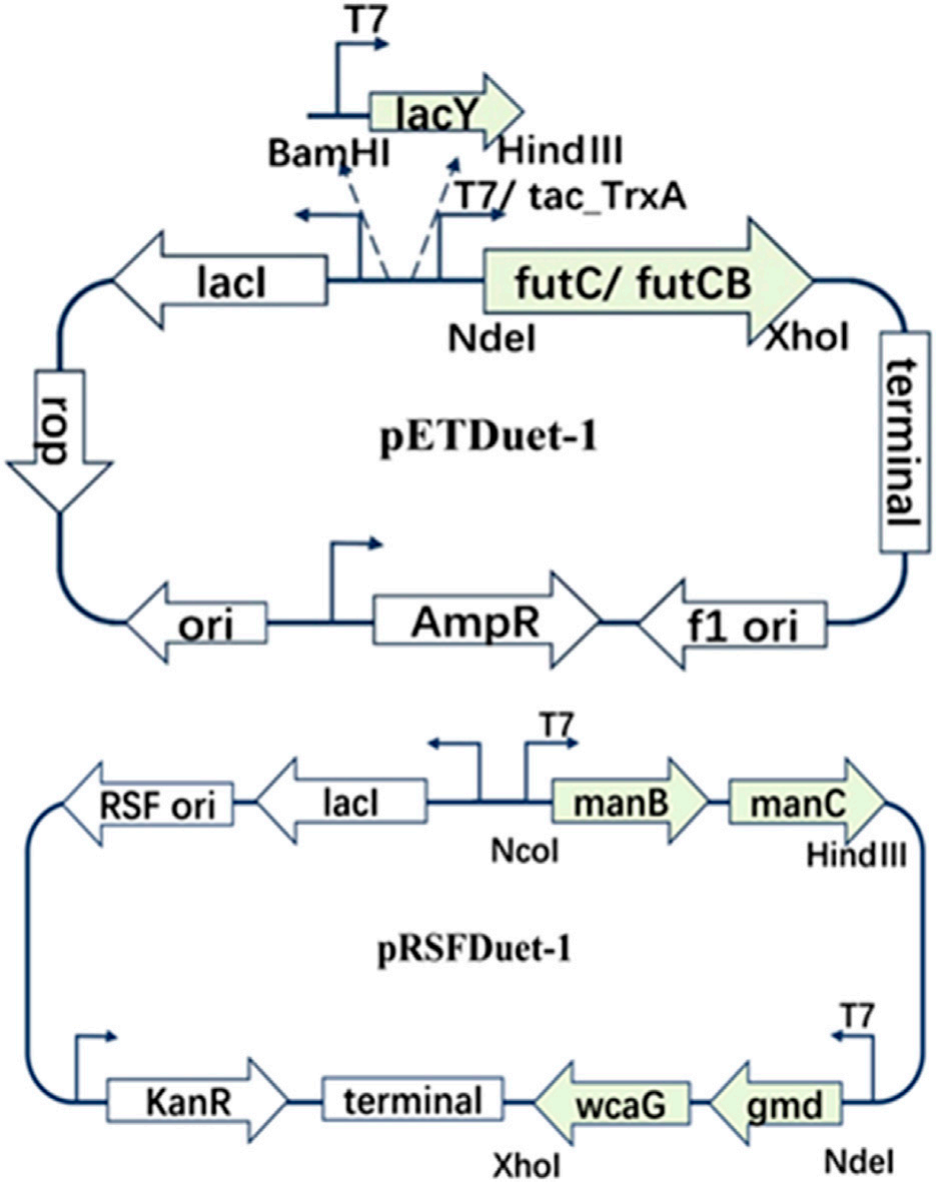
Co-expression of different plasmid pETDuet-1 and pRSFDuet-1 biosynthetic cassete contains different genes.

To enable the biosynthesis of 2′-FL in *E. coli*, the final fucosylation step, catalyzed by α1,2-fucosyltransferase, was incorporated. This enzyme uses lactose and GDP-L-fucose as substrates. The α1,2-fucosyltransferases from *Helicobacter pylori* (FutC) and *Bacillus cereus* (FutCB) were selected for this purpose (Xu et al., 2021). FutC has been reported to be suitable for the construction of 2′-FL synthesis pathway in Gram-negative bacteria, while FutCB was only utilized in *S. cerevisiae* before. Lactose can be transported into *E. coli* by β-galactoside permease (*lacY*), a membrane protein which embedded in the plasma membrane. However, lactose can also be hydrolyzed by β-galactosidase (*lacZ*), so the inactivation of β-galactosidase in *E. coli* C43 (DE3) strain was firstly achieved with CRISPR-Cas9 (named CΔZ). GDP-L-fucose, another important precursor in the 2′-FL synthesis pathways, which can be generated by the *de novo* pathway in *E. coli via* five enzymes mannose-6-phosphate isomerase (manA), phosphomannomutase (manB), mannose-1-phosphate guanosyltransferase (manC), GDP-D-mannose-4,6-dehydratase (gmd) and GDP-L-fucose synthase (wcaG). These five enzymes can gradually catalyze the metabolic intermediate fructose-6-P of glycolysis into mannose-6-P, mannose-1-P, GDP-D-mannose, GDP-4-dehydro-6-deoxymannose, and finally into GDP-L-fucose (Figure 1). Therefore, in order to increase the accumulation of GDP-L-fucose, the co-expression of endogenous genes manB, manC, gmd, and wcaG were performed in *E. coli* (Figure 2).

### 3.2 Optimizing the expression of α1,2-fucosyltransferase

**FIGURE 3:**
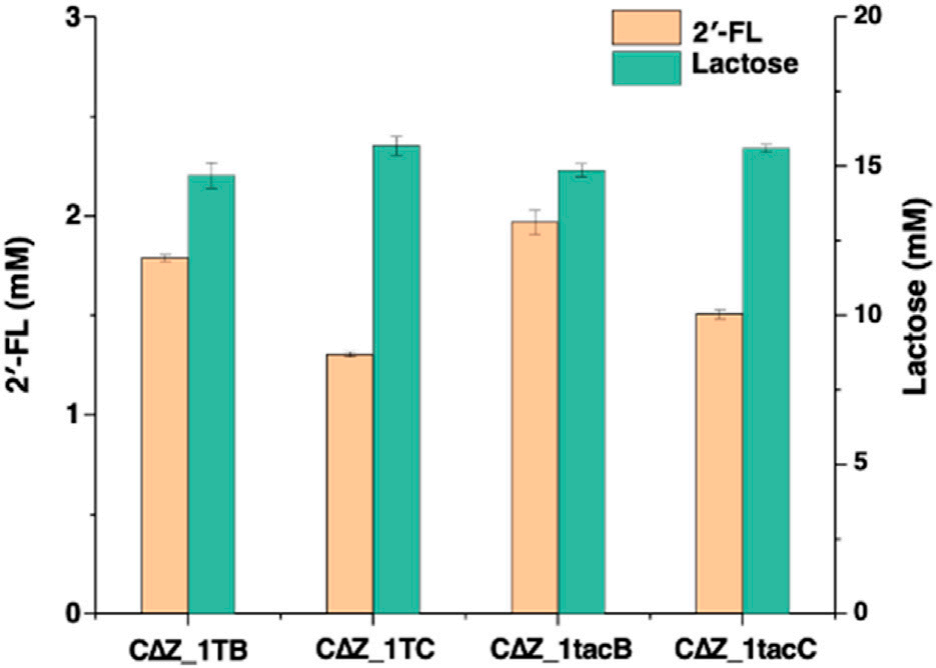
Engineered *Escherichia coli* strains express combinations of T7/Ptac promoters with TrxA-FutC/FutCB fusion proteins. The detection of 2′-FL and lactose by LC-MS was performed for the following strains: CΔZ_1TB, which harbored the plasmids pRS-CBGW and pET-T7FutCB; CΔZ_1TC, containing pRS-CBGW and pET-T7FutC; CΔZ_1tacB, with plasmids pRS-CBGW and pET-tac_TrxA_FutCB; and CΔZ_1tacC, carrying pRS-CBGW and pET-tac_TrxA_FutC.

To optimize the expression of α1,2-fucosyltransferase in *E. coli*, different combinations of promoters (T7 and P_tac_ with fusion protein TrxA) and α1,2-fucosyltransferases (FutC and FutCB) were elucidated in *lacZ* inactive strain CΔZ_1 (CΔZ harboring plasmids pRS-CBGW), resulting in the four engineered strains CΔZ_1TB, CΔZ_1TC, CΔZ_1tacB and CΔZ_1tacC (Supplementary Table S1). Batch fermentations of the engineered strains were performed in 500 mL baffled shake flasks to evaluate their 2′-FL production capabilities. After 72 h of cultivation, the 2′-FL yields were determined as follows: CΔZ_1 TB (1.788 mM), CΔZ_1 TC (1.303 mM), CΔZ_1tacB (1.970 mM), and CΔZ_1tacC (1.506 mM). The strain CΔZ_1tacB, carrying pRS-CBGW and pET-tac_TrxA_FutCB, exhibited the highest 2′-FL yield with a conversion efficiency of 11.66% (2′-FL mol per lactose mol) (Figure 3). Furthermore, the initial concentration of lactose was about 16.9 mM.

Meanwhile, the advantages of FutCB and TrxA can be observed when we compare the substrate conversion of CΔZ_1TB, CΔZ_1TC, CΔZ_1tacB and CΔZ_1tacC. The combination of promoter tac and TrxA has been reported in Lin’s report for the synthesis of 2′-FL, with a 1.7-fold improvement compared with the untagged (Lin et al., 2022). Since the gene transcription and translation in *E. coli* often occur simultaneously, the quick transcription with T7 promoter is likely to cause protein misfolding. To solve this problem, the use of a low transcription promoter and fusion protein is helpful.

**FIGURE 4:**
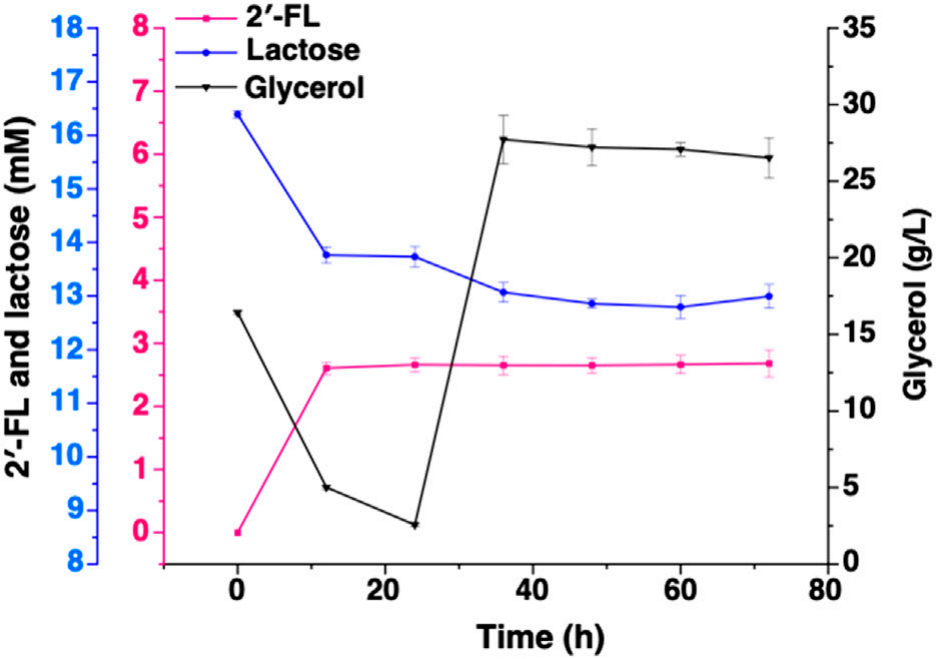
The introduction of the *lacY* (lactose transporter). The *de novo* biosynthetic pathway for 2′-FL was assessed in the *Escherichia coli* C43 (DE3) strain, specifically the engineered CΔZ_1YtacB, which carried the plasmids pRS-CBGW and pET-T7*lacY*-tac_TrxA_FutCB.

In addition, since intracellular lactose cannot be monitored in real time, the low substrate concentration may also be an important factor limiting the synthesis of 2′-FL. Therefore, we inserted the endogenous gene *lacY* of *E. coli* into the plasmid pET-tac_TrxA_FutCB/FutC and pET-T7FutCB/FutC to promote the transport of lactose. After 64-h batch fermentation, strain CΔZ_1YtacB harboring pRS-CBGW and pET-T7*lacY*-tac_TrxA_FutCB achieved the highest amount of 2′-FL synthesis, with a conversion of significantly higher than 15.88%. Results show that the overexpression of *lacY* increased the yield of 2′-FL (Figure 4).

### 3.3 Genetic modifications to enhance GDP-L-fucose accumulation in *Escherichia coli* for 2′-FL synthesis

In *E. coli*, GDP-L-fucose is used as a source of L-fucose in the biosynthesis of colanic acid (CA), a key exopolysaccharide found in the Enterobacteriaceae family. CA is produced by polymerizing d-glucose (Glc), d-galactose (Gal), l-fucose (Fuc), and d-glucuronic acid (GlcA) in a 1:2:2:1 M ratio, with further modifications involving non-stoichiometric acetyl and pyruvate groups. The synthesis of CA is thought to play a role in the bacterium’s response to environmental stressors. The *wcaJ*, *wcaE*, and *wcaI* genes are involved in initiating CA biosynthesis, with fucose being incorporated during the second and third steps of the process.

**FIGURE 5:**
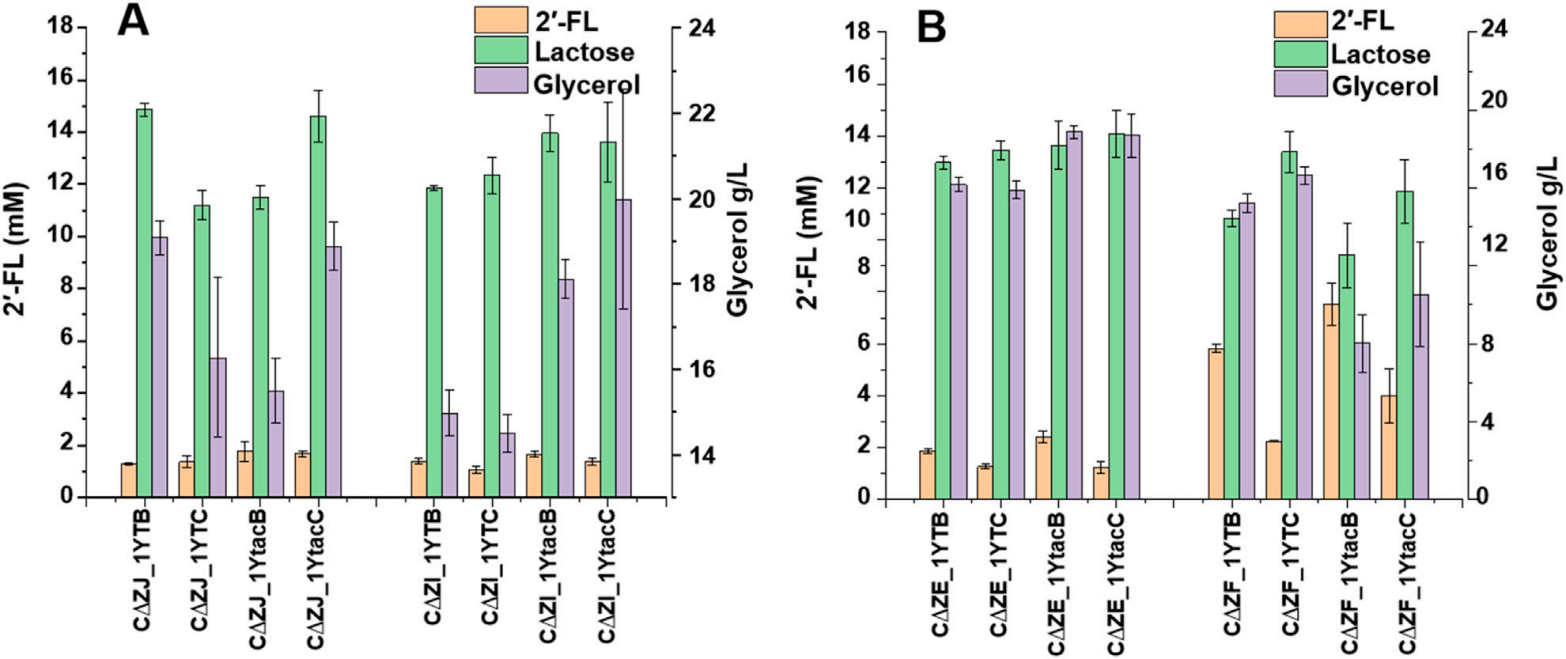
The production of 2′-FL and lactose through the *de novo* pathway in engineered *Escherichia coli* C43 (DE3) strains was compared, with an emphasis on glycerol consumption. The strains used in the study included 1YTB, which contains plasmids pRS-CBGW and pET-T7*lacY*-T7FutCB; 1YTC, containing plasmids pRS-CBGW and pET-T7*lacY*-T7futC; 1YtacB, with plasmids pRS-CBGW and pET-T7*lacY*-tac_TrxA_futCB; and 1YtacC, carrying plasmids pRS-CBGW and pET-T7*lacY*-tac_TrxA_FutC. **(A)**: knock out of the genes *wcaJ* and *wcaI* respectively to produce strains CΔZE and CΔZI; **(B)**: knock out of the genes *wcaE* and *waaF* respectively to produce strains CΔZE and CΔZF.

The accumulation of GDP-L-fucose in engineered strain determines the total production of 2′-FL. Four genes, manB, manC, gmd, and wcaG, were overexpressed in the *lacZ* inactive *E. coli* strain, although the final 2′-FL production obtained from batch fermentation was not enough for industrial synthesis. To break the consumption pathway of GDP-L-fucose, *wcaE, wcaJ,* and *wcaI* were knocked out respectively, generating strains CΔZE, CΔZJ, and CΔZI. These deletions led to increase in 2′-FL synthesis, as shown in Figure 5. However, the *wcaJ* deletion resulted in less significant production than anticipated, suggesting that alternative metabolic pathways may compensate for its inactivation. 2′-FL synthesis was observed after 72 h of fermentation in a 500 mL baffled shake flask using these strains.

GDP-L-fucose can be effectively accumulated in *E. coli* with double deletion of *waaF* and *wcaJ* genes in Lee’s report. Gene *waaF* encodes ADP-heptose: LPS heptosyltransferase II, which is involved in the LPS pathway and is related to the synthesis of CA. In this report, the GDP-L-fucose accumulated by the double deletion *E. coli* strain was improved 1.36 times (Lee et al., 2021).

Therefore, we inactivated the *waaF* gene, and the batch fermentation results are shown in Figure 5. Compared with CΔZ, the CΔZF strain with double inactivation of *lacZ* and *waaF* exhibited a 2-fold increase in shake flask fermentation across different plasmid combinations. Among them, the titer of shake flask fermentation of strain CΔZF-1YtacB was 7.52 mM, and the conversion rate was 44.50%. Compared with CΔ*Z*-1YtacB (15.88%), the conversion rate was increased by 2.8 times. The titer of shake flask fermentation of strain CΔZF_1YTB was 5.81 mM, and the conversion rate was 34.38%. Compared with CΔZ_1YTB (4.9%), the conversion rate was increased by 7.19 times. The titer of shake flask fermentation of strain CΔZF-1YtacC was 3.99 mM, and the conversion rate was 23.6%. Compared with CΔZ-1YtacC (10.35%), the conversion rate was increased by 2.28 times.

It has been reported that the deletion of *waaF* in *E. coli* leads to the accumulation of CA (Han et al., 2021). This is because the ADP-heptose: LPS heptosyltransferase II encoded by *waaF* uses UDP-D-glucose to synthesize Lipid A-core, and UDP-D-glucose is the first precursor for CA synthesis. Therefore, in the *E. coli* C43 (DE3) strain, instead of knocking out genes such as *wcaJ* to cut off the consumption path of GDP-L-fucose, knocking out *waaF* to promote the flow of UDP-D-glucose to the direction of CA synthesis, thereby promoting the large-scale synthesis of GDP-L-fucose, is more effective.

### 3.4 Production of 2′-FL with fed batch fermentation using the constitutive promoters

For the ultimate goal of large-scale production of 2′-FL, compared with inducible promoters, constitutive promoters have the advantages of continuous expression, simple control (no need for additional inducers), low cost, reduced metabolic burden, and stability. Based on the engineered strain CΔZF-1YtacB, we further reconstructed the genes manB, manC, gmd, and wcaG that needed to be overexpressed onto plasmid pET-BCGW-*zwf* containing the constitutive promoter J23118.

Additionally, the regulator of capsule synthesis (Rcs) system is an unconventional two-component system commonly found in many Gram-negative bacteria. It includes the response regulator RcsB, the auxiliary regulator RcsA, the transmembrane sensor kinase RcsC, the transmembrane protein RcsD, and the membrane-bound lipoprotein RcsF (Wang et al., 2020). The RcsB or RcsA-RcsB dimer activates the transcription of the wca operon, which is involved in colanic acid (CA) biosynthesis. Based on the engineered strain CΔZF-1YtacB, we further reconstructed the genes FutCB, *fkp*, RcsA, and RcsB for overexpression on the plasmid pAC-FFGR-*lacY*, which contains the constitutive promoter J23111. The plasmids pET-BCGW-*zwf* and pAC-FFGR-*lacY* were transformed into the CΔZF strain, and shake flask fermentation was performed, resulting in a yield of 15 g/L after 48 h of fermentation.

**FIGURE 6:**
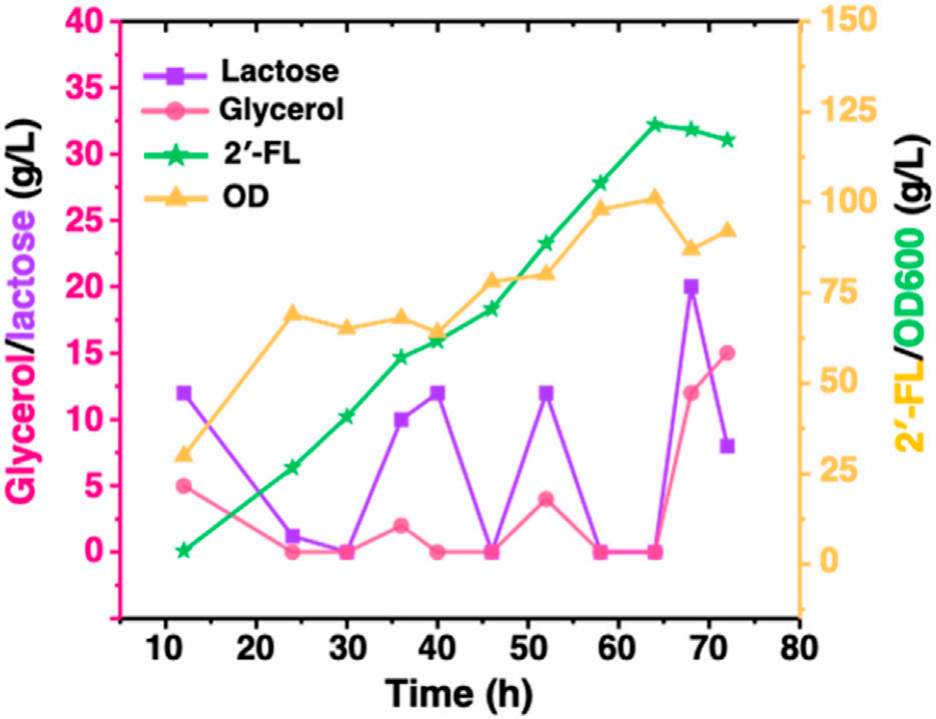
Production of 2′-FL by fed-batch cultivation of strain CΔZF (harbor two plasmids: pET-BCGW-*zwf* and pAC-FFGR-*lacY*). Biomass conversion rate and production of 2′-FL using Fed-batch fermentation. The pink and purple lines show the consumption of glycerol and lactose as a carbon source while the orange line indicates the production of 2′-FL reaching to a maximum of 121 g/L after 64 h.

To demonstrate the performance of engineered strain CΔZF harboring pET-BCGW-*zwf* and pAC-FFGR-*lacY* in large-scale 2′-FL production, we performed further fed-batch fermentation in a 10 L fermenter. 5 L of fermentation medium was used as the initial medium for growth, and 72 h fed-batch fermentation was carried out under optimized conditions in a 10 L fermenter. It can be found that during the entire fermentation process, the biomass showed an overall increasing trend and did not decrease significantly. After 46 h of fermentation, the OD_600_ reached more than 80, indicating that the double gene knock-out ensured the vitality of the host bacteria, and that our production system has a certain stability. As shown in Figure 6, the accumulation of 2′-FL is proportional to the biomass. This is because the constitutive promoter used in this study allows the FutCB gene to be stably and continuously expressed, and the accumulation of 2′-FL does not show the lag period. At the same time, the constitutive promoter also ensures that the gene is not affected by environmental factors or host status, and no additional inducer is required, which is more suitable for industrial production. With the consumption of lactose, the product continues to accumulate and reaches a peak at 64 h, at which time and space the titer and yield is 121.4 g/L and 1.90 g/L/h, exceeding the results reported (Wang et al., 2020).

## 4 Conclusion

This study demonstrated the first use of *B. cereus* FutCB for *de novo* biosynthesis of 2′-FL in *E. coli* based microbial cell factory. Strategic inactivation of *lacZ* and *waaF* together with the overexpression of gmd, manB, wcaG and manC was effective for improving the accumulation of the required substrates, lactose and GDP-L-fucose. Biosynthesis of 2′-FL was significantly stabilized with the use of constitutive promoter especially under the fed-batch fermentation process. The production titer achieved in this study 121.4 g/L is higher than previously reported, representing a key breakthrough in the microbial production of 2′-FL. These findings provide a foundation for future industrial applications, also further optimization may result in even higher production yields.

## Data Availability

The original contributions presented in the study are included in the article/Supplementary Material, further inquiries can be directed to the corresponding authors.

## References

[B1] Agoston K. Hederos M. J. Bajza I. Dekany G. (2019). Kilogram scale chemical synthesis of 2′-fucosyllactose. Carbohydr. Res. 476, 71–77. 10.1016/j.carres.2019.03.006 30921739

[B2] Albermann C. Distler J. Piepersberg W. (2000). Preparative synthesis of GDP-β-L-fucose by recombinant enzymes from enterobacterial sources. Glycobiology 10 (9), 875–881. 10.1093/glycob/10.9.875 10988249

[B3] Albermann C. Piepersberg W. Wehmeier U. F. (2001). Synthesis of the milk oligosaccharide 2′-fucosyllactose using recombinant bacterial enzymes. Carbohydr. Res. 334 (2), 97–103. 10.1016/s0008-6215(01)00177-x 11502265

[B4] Baumgärtner F. Seitz L. Sprenger G. A. Albermann C. (2013). Construction of *Escherichia coli* strains with chromosomally integrated expression cassettes for the synthesis of 2′-fucosyllactose. Microb. Cell Factories 12, 40–13. 10.1186/1475-2859-12-40 PMC3655002 23635327

[B5] Chen S. Li G. Wu N. Guo X. Liao N. Ye X. (2013). Sulfation pattern of the fucose branch is important for the anticoagulant and antithrombotic activities of fucosylated chondroitin sulfates. Biochimica Biophysica Acta (BBA)-General Subj. 1830 (4), 3054–3066. 10.1016/j.bbagen.2013.01.001 23313164

[B6] Deng J. Chen C. Gu Y. Lv X. Liu Y. Li J. (2019a). Creating an *in vivo* bifunctional gene expression circuit through an aptamer-based regulatory mechanism for dynamic metabolic engineering in Bacillus subtilis. Metab. Eng. 55, 179–190. 10.1016/j.ymben.2019.07.008 31336181

[B7] Deng J. Gu L. Chen T. Huang H. Yin X. Lv X. (2019b). Engineering the substrate transport and cofactor regeneration systems for enhancing 2′-fucosyllactose synthesis in Bacillus subtilis. ACS Synth. Biol. 8 (10), 2418–2427. 10.1021/acssynbio.9b00314 31550146

[B8] Efsa Panel on Nutrition N. F. Allergens F. Turck D. Castenmiller J. De Henauw S. HirschErnst K. I. (2019). Safety of 2’fucosyllactose/difucosyllactose mixture as a novel food pursuant to Regulation (EU) 2015/2283. EFSA J. 17 (6), e05717. 10.2903/j.efsa.2019.5717 32626339 PMC7009083

[B9] EFSA Panel on Nutrition, Novel Foods and Food Allergens (NDA), Turck D. Bohn T. Castenmiller J. De Henauw S. Hirsch-Ernst K. I. Maciuk A. (2022). Safety of 2′‐fucosyllactose (2’FL) produced by a derivative strain (APC199) of Corynebacterium glutamicum ATCC 13032 as a novel food pursuant to Regulation (EU) 2015/2283. EFSA J. 20 (12), e07647. 10.2903/j.efsa.2022.7647 36531695 PMC9749449

[B10] Goormans A. R. Snoeck N. Decadt H. Vermeulen K. Peters G. Coussement P. (2020). Comprehensive study on *Escherichia coli* genomic expression: does position really matter? Metab. Eng. 62, 10–19. 10.1016/j.ymben.2020.07.007 32795614

[B11] Han H. M. Kim I. J. Yun E. J. Lee J. W. Cho Y. Jin Y.-S. (2021). Overproduction of exopolysaccharide colanic acid by *Escherichia coli* by strain engineering and media optimization. Appl. Biochem. Biotechnol. 193, 111–127. 10.1007/s12010-020-03409-4 32820352

[B12] Hollands K. Baron C. M. Gibson K. J. Kelly K. J. Krasley E. A. Laffend L. A. (2019). Engineering two species of yeast as cell factories for 2′-fucosyllactose. Metab. Eng. 52, 232–242. 10.1016/j.ymben.2018.12.005 30557615

[B13] Jiang Y. Chen B. Duan C. Sun B. Yang J. Yang S. (2015). Multigene editing in the *Escherichia coli* genome via the CRISPR-Cas9 system. Appl. Environ. Microbiol. 81 (7), 2506–2514. 10.1128/AEM.04023-14 25636838 PMC4357945

[B14] Lee H. J. Shin D. J. Han K. Chin Y. W. Park J. P. Park K. (2022). Simultaneous production of 2′‐fucosyllactose and difucosyllactose by engineered *Escherichia coli* with high secretion efficiency. Biotechnol. J. 17 (3), 2100629. 10.1002/biot.202100629 35073455

[B15] Lee J. W. Kwak S. Liu J.-J. Yun E. J. Jin Y.-S. (2021). 2′-Fucosyllactose production in engineered *Escherichia coli* with deletion of waaF and wcaJ and overexpression of FucT2. J. Biotechnol. 340, 30–38. 10.1016/j.jbiotec.2021.08.007 34450187

[B16] Li C. Wu M. Gao X. Zhu Z. Li Y. Lu F. (2020). Efficient biosynthesis of 2′-fucosyllactose using an *in vitro* multienzyme cascade. J. Agric. Food Chem. 68 (39), 10763–10771. 10.1021/acs.jafc.0c04221 32856455

[B17] Lin L. Gong M. Liu Y. Li J. Lv X. Du G. (2022). Combinatorial metabolic engineering of *Escherichia coli* for *de novo* production of 2′-fucosyllactose. Bioresour. Technol. 351, 126949. 10.1016/j.biortech.2022.126949 35257882

[B18] Liu Y. Zhu Y. Wan L. Chen R. Zhang W. Mu W. (2022). High-level *de novo* biosynthesis of 2′-fucosyllactose by metabolically engineered *Escherichia coli* . J. Agric. Food Chem. 70 (29), 9017–9025. 10.1021/acs.jafc.2c02484 35834320

[B19] Ni Z. Li Z. Wu J. Ge Y. Liao Y. Yuan L. (2020). Multi-path optimization for efficient production of 2′-fucosyllactose in an engineered *Escherichia coli* C41 (DE3) derivative. Front. Bioeng. Biotechnol. 8, 611900. 10.3389/fbioe.2020.611900 33425876 PMC7793955

[B20] Park B. S. Choi Y. H. Kim M. W. Park B. G. Kim E. J. Kim J. Y. (2022). Enhancing biosynthesis of 2'Fucosyllactose in *Escherichia coli* through engineering lactose operon for lactose transport and α1, 2Fucosyltransferase for solubility. Biotechnol. Bioeng. 119 (5), 1264–1277. 10.1002/bit.28048 35099812

[B21] Park B. S. Yoon J. Lee J. M. Cho S. H. Choi Y. Cho B. K. (2025). Metabolic engineering of Priestia megaterium for 2’-fucosyllactose production. Microb. Cell Factories 24 (1), 2. 10.1186/s12934-024-02620-w PMC11699682 39754105

[B22] Qian D. Zhang C. Deng C. Zhou M. Fan L. Zhao L. (2023). *De novo* biosynthesis of 2′-fucosyllactose in engineered Pichia pastoris. Biotechnol. Lett. 45 (4), 521–536. 10.1007/s10529-023-03357-z 36790735

[B23] Qiao J. Zhan Y. Tan X. Liu Y. Hu X. Wang X. (2021). Colanic acid: biosynthetic overproduction by engineering *Escherichia coli* and physical property characterization. J. Agric. Food Chem. 69 (46), 13881–13894. 10.1021/acs.jafc.1c04823 34763421

[B24] Shi R. Zhang D.-y. Gu Y.-h. Jiang Z.-q. Yang S.-q. (2021). “Direct evolution of α-L-fucosidase from Pedobacter sp,” in And its application in the synthesis of 2'-fucosyllactose. 10.1007/s00253-020-10630-y

[B25] Shi R. Jiang Z. (2020). Enzymatic synthesis of 2'-fucosyllactose: advances and perspectives. Synthetic Biol. J. 1 (4), 481. 10.1021/acs.jafc.0c04221

[B26] Sun X. Peng Z. Li C. Zheng Y. Zong J. Lu F. (2023). Combinatorial metabolic engineering and tolerance evolving of *Escherichia coli* for high production of 2′-fucosyllactose. Bioresour. Technol. 372, 128667. 10.1016/j.biortech.2023.128667 36702325

[B27] Vandenplas Y. Berger B. Carnielli V. P. Ksiazyk J. Lagström H. Sanchez Luna M. (2018). Human milk oligosaccharides: 2′-fucosyllactose (2′-FL) and lacto-N-neotetraose (LNnT) in infant formula. Nutrients 10 (9), 1161. 10.3390/nu10091161 30149573 PMC6164445

[B28] Wan L. Zhu Y. Chen G. Luo G. Zhang W. Mu W. (2021). Efficient production of 2′-fucosyllactose from L-fucose via self-assembling multienzyme complexes in engineered *Escherichia coli* . ACS Synth. Biol. 10 (10), 2488–2498. 10.1021/acssynbio.1c00102 34415729

[B29] Wang C. Zhang H. Wang J. Chen S. Wang Z. Zhao L. (2020). Colanic acid biosynthesis in *Escherichia coli* is dependent on lipopolysaccharide structure and glucose availability. Microbiol. Res. 239, 126527. 10.1016/j.micres.2020.126527 32590169

[B30] Xu M. Meng X. Zhang W. Shen Y. Liu W. (2021). Improved production of 2′-fucosyllactose in engineered *Saccharomyces cerevisiae* expressing a putative α-1, 2-fucosyltransferase from Bacillus cereus. Microb. Cell Factories 20, 165–213. 10.1186/s12934-021-01657-5 PMC8381501 34425826

[B31] Yu S. Liu J.-J. Yun E. J. Kwak S. Kim K. H. Jin Y.-S. (2018). Production of a human milk oligosaccharide 2′-fucosyllactose by metabolically engineered *Saccharomyces cerevisiae* . Microb. Cell Factories 17 (1), 101–110. 10.1186/s12934-018-0947-2 PMC6020385 29950173

[B32] Zeuner B. Meyer A. S. (2020). Enzymatic transfucosylation for synthesis of human milk oligosaccharides. Carbohydr. Res. 493, 108029. 10.1016/j.carres.2020.108029 32445980

[B33] Zhang Q. Liu Z. Xia H. Huang Z. Zhu Y. Xu L. (2022). Engineered Bacillus subtilis for the *de novo* production of 2′-fucosyllactose. Microb. Cell Factories 21 (1), 110. 10.1186/s12934-022-01838-w PMC9164505 35655274

[B34] Zhang Q. Xu X. Zhang W. Huang Z. Wu Y. Liu Y. (2025). *De novo* 2′-fucosyllactose biosynthesis using glucose as the sole carbon source by multiple engineered Bacillus subtilis. Metab. Eng. 88, 85–93. 10.1016/j.ymben.2024.12.004 39694455

[B35] Zhang Z. Li Y. Wu M. Gao Z. Wu B. He B. (2023). Identification and characterization of a novel α-L-fucosidase from Enterococcus gallinarum and its application for production of 2′-fucosyllactose. Int. J. Mol. Sci. 24 (14), 11555. 10.3390/ijms241411555 37511315 PMC10380807

[B36] Zhou W. Jiang H. Liang X. Qiu Y. Wang L. Mao X. (2022). Discovery and characterization of a novel α-L-fucosidase from the marine-derived Flavobacterium algicola and its application in 2′-fucosyllactose production. Food Chem. 369, 130942. 10.1016/j.foodchem.2021.130942 34479010

[B37] Zhu Y. Zhao M. Wang H. Zhu Y. Mu W. (2025). Metabolic engineering of *Escherichia coli* BL21 (DE3) cocultured with glucose and xylose for efficient production of 2′-fucosyllactose. Bioresour. Technol. 419, 132062. 10.1016/j.biortech.2025.132062 39832618

