# Supplementary material for: Intensification of 2′-Fucosyllactose biosynthesis pathway by using a novel fucosyltransferase from Bacillus cereus
Source: Front Bioeng Biotechnol. 2025 Apr 30;13:1569597. doi: 10.3389/fbioe.2025.1569597 (PMC12075129; doi:10.3389/fbioe.2025.1569597)
Supplement: Supplementary file 1 [file Table1.docx]

Supplementary Material

Intensification of 2'-Fucosyllactose Biosynthesis pathway by using a novel fucosyltransferase from *Bacillus cereus*

Kainuo Zhang ^1+^, Miaomiao Gao ^1+^, Chenqi Cao ^1^, Mengxin Zhang ^1^, Waqar Ahmad ^1^, Ahmed Rady ^2^, Badr Aldahmash ^2^, Tianze Zhu ^3^, Shahin Shah Khan ^1^*, Luo Liu ^1^*

^1^ College of Life Science and Technology, Beijing University of Chemical Technology, No. 15 East Road of North Third Ring Road, Beijing 100029, China

^2^ Department of Zoology, College of Science, King Saud University, P. O. Box 2455, Riyadh 11451, Saudi Arabia

^3^ Beijing Zeno Biotechnology Development Co. Ltd, Tianrong West Road, Biological Medicine Industrial Park, Daxing District, Beijing

+ Both are equal authors

*** Correspondence:**Shahin Shah Khan and Luo Liu
shahinshah@buct.edu.cn, liuluo@mail.buct.edu.cn

**Table S1. Metabolic engineered strains for 2'-FL production through *de novo* pathway.**

| **Strains** | **Relevant description** | **Reference** |
| --- | --- | --- |
| C43(DE3) | *E. coli* C43(DE3) | WEIDI |
| CΔZ | *E. coli* C43(DE3) Δ*lacZ* | This study |
| CΔZ_1 | CΔZ harboring plasmids pRS-CBGW | This study |
| CΔZ_1TB | CΔZ harboring plasmids pRS-CBGW and pET-T7FutCB | This study |
| CΔZ_1TC | CΔZ harboring plasmids pRS-CBGW and pET-T7FutC | This study |
| CΔZ_1tacB | CΔZ harboring plasmids pRS-CBGW and pET-tac_TrxA_FutCB | This study |
| CΔZ_1tacC | CΔZ harboring plasmids pRS-CBGW and pET-tac_TrxA_FutC | This study |
| CΔZ_1YTB | CΔZ harboring plasmids pRS-CBGW and pET-T7*lacY*-T7FutCB | This study |
| CΔZ_1YTC | CΔZ harboring plasmids pRS-CBGW and pET-T7*lacY*-T7FutC | This study |
| CΔZ_1YtacB | CΔZ harboring plasmids pRS-CBGW and pET-T7*lacY*-tac_TrxA_FutCB | This study |
| CΔZ_1YtacC | CΔZ harboring plasmids pRS-CBGW and pET-T7*lacY*-tac_TrxA_FutC | This study |
| CΔZE | *E. coli* C43(DE3)Δ*lacZ* Δ*wcaE* | This study |
| CΔZJ | *E. coli* C43(DE3) Δ*lacZ*Δ*wcaJ* | This study |
| CΔZI | *E. coli* C43(DE3) Δ*lacZ*Δ*wcaI* | This study |
| CΔZE_1YTB | CΔZE harboring plasmids pRS-CBGW and pET-T7*lacY*-T7FutCB | This study |
| CΔZE_1YTC | CΔZE harboring plasmids pRS-CBGW and pET-T7l*acY*-T7FutC | This study |
| CΔZE_1YtacB | CΔZE harboring plasmids pRS-CBGW and pET-T7lacY-tac_TrxA_FutCB | This study |
| CΔZE_1YtacC | CΔZE harboring plasmids pRS-CBGW and pET-T7*lacY*-tac_TrxA_FutC | This study |
| CΔZJ_1YTB | CΔZJ harboring plasmids pRS-CBGW and pET-T7*lacY*-T7FutCB | This study |
| CΔZJ_1YTC | CΔZJ harboring plasmids pRS-CBGW and pET-T7*lacY*-T7FutC | This study |
| CΔZJ_1YtacB | CΔZJ harboring plasmids pRS-CBGW and pET-T7*lacY*-tac_TrxA_FutCB | This study |
| CΔZJ_1YtacC | CΔZJ harboring plasmids pRS-CBGW and pET-T7*lacY*-tac_TrxA_FutC | This study |
| CΔZI_1YTB | CΔZI harboring plasmids pRS-CBGW and pET-T7*lacY*-T7FutCB | This study |
| CΔZI_1YTC | CΔZI harboring plasmids pRS-CBGW and pET-T7*lacY*-T7FutC | This study |
| CΔZI_1YtacB | CΔZI harboring plasmids pRS-CBGW and pET-T7*lacY*-tac_TrxA_FutCB | This study |
| CΔZI_1YtacC | CΔZI harboring plasmids pRS-CBGW and pET-T7*lacY*-tac_TrxA_FutC | This study |
| CΔZF | *E. coli* C43(DE3)Δ*lacZ* Δ*waaF* | This study |
| CΔZF_1YTB | CΔZF harboring plasmids pRS-CBGW and pET-T7*lacY*-T7FutCB | This study |
| CΔZF_1YTC | CΔZF harboring plasmids pRS-CBGW and pET-T7*lacY*-T7FutC | This study |
| CΔZF_1YtacB | CΔZF harboring plasmids pRS-CBGW and pET-T7*lacY*-tac_TrxA_FutCB | This study |
| CΔZF_1YtacC | CΔZF harboring plasmids pRS-CBGW and pET-T7*lacY*-tac_TrxA_FutC | This study |
| C2 | CΔZF harboring plasmids pET-BCGW-*zwf* and pAC-FFGR-*lacY* | This study |

**Table S2. Primers used in this study**

| **Primers** | **Sequence** |
| --- | --- |
| **Primers for the construction of specific pTargetF** | |
| *LacZ*-sgRNA-F | **TCCTAGGTATAATACTAGT**GAGTGTGATCATCTGGTCGC**GTTTTAGAGCTAGAAATAGC** |
| *LacZ*-sgRNA-R | ACTAGTATTATACCTAGGACTGAGCTAGCTGTCAAG |
| *waaF*-sgRNA-F | **TCCTAGGTATAATACTAGT**ATTCCTATGCCTCTCGGTCA**GTTTTAGAGCTAGAAATAGCAAGT** |
| *waaF-s*gRNA-R | ACTAGTATTATACCTAGGACTGAGCTAGCTGTCAAG |
| *wcaj*-sgRNA-F | **TCCTAGGTATAATACTAGT**GTCAGCACATTGATAAACTG**GTTTTAGAGCTAGAAATAGC** |
| *wcaj*-sgRNA-R | ACTAGTATTATACCTAGGACTGAGCTAGCTGTCAAG |
| *nudK*-sgRNA-F | **TCCTAGGTATAATACTAGT**ACCGAGCACCACATAGTGAG**GTTTTAGAGCTAGAAATAGC** |
| *nudK-s*gRNA-R | ACTAGTATTATACCTAGGACTGAGCTAGCTGTCAAG |
| *nudD*-sgRNA-F | **TCCTAGGTATAATACTAGT**ACCGAGCACCACATAGTGAG**GTTTTAGAGCTAGAAATAGC** |
| *nudD*-sgRNA-R | ACTAGTATTATACCTAGGACTGAGCTAGCTGTCAAG |
| *wcaI*-sgRNA-F | **TCCTAGGTATAATACTAGT**AAGCGTCATACGATTGCAGC**GTTTTAGAGCTAGAAATAGC** |
| *wcaI*-sgRNA-R | ACTAGTATTATACCTAGGACTGAGCTAGCTGTCAAG |
| *wcaE*-sgRNA-F | **TCCTAGGTATAATACTAGT**GCAGCCAAAATGTATAAAGC**GTTTTAGAGCTAGAAATAGC** |
| *wcaE*-sgRNA-R | ACTAGTATTATACCTAGGACTGAGCTAGCTGTCAAG |
| **Primers for the amplification of homologous arms of the target gene, upstream (US) and downstream (DS)** | |
| *LacZ*-US-F | ACGCGAAATACGGGCAGACA |
| *LacZ*-US-R | CTACGTCTGAACGTCGGGTCTGCGCTGCGGG |
| *LacZ-*DS-F | CGACGTTCAGACGTAGTGTGACG |
| *LacZ-*DS-R | GCCAGGACAGTCGTTTGCC |
| *waaF-*US-F | CGCAGATTGTTGGCTTCCGT |
| *waaF-*US-R | GTAACAATAGCGCGTTGCAACCCAAGACGGGCCGAT |
| *waaF-*DS-F | CAACGCGCTATTGTTACAAGAGGAAGC |
| *waaF*-DS-R | TTTTGGCAAACAGTTCGCGG |
| *wcaj*-US-F | CATCGTTAATCTCTATGGTG |
| *wcaj*-US-R | TACTCAAGGTCGAACTCGACGTCGACCAGTTGTTGCAGATTG |
| *wcaj-*DS-F | CAATCTGCAACAACTGGTCGACGTCGAGTTCGACCTTGAGTA |
| *wcaj-*DS-R | CAGCACGTCACCAATGAGATC |
| *nudK*-US-F | GAATCATCTGCAAAAACGCGGTGAT |
| *nudK*-US-R | AGACGGCGAAGTTATCCCCGACAAATCCGATTGAGCCG |
| *nudK-*DS-F | GGATAACTTCGCCGTCTTTGCG |
| *nudK-*DS-R | GGAAAGCCAGTACCGTTACCAGAC |
| *nudD*-US-F | GCTACGTTCGAACGCCGTC |
| *nudD*-US-R | ACGAAACGCTGGAAGCCCGGCATTAACTACTCGCCGG |
| *nudD-*DS-F | GGCTTCCAGCGTTTCGTCTTTC |
| *nudD-*DS-R | TTACGTCAGGAAGACTTTGCCAC |
| *wcaI*-US-F | GAGCGTGCGTTCGGCATATTC |
| *wcaI*-US-R | CGAAGCTCTCTGGTGCGCACTGCTGAAGATGGGC |
| *wcaI-*DS-F | CACCAGAGAGCTTCGCCAGC |
| *wcaI-*DS-R | GTCTACGGCATTAACTACTCGCCG |
| *wcaE*-US-F | GTCTTTGAGGTAGTACGTTGGC |
| *wcaE*-US-R | ACGCTTTGTCAGCGACTCAATGGCCTGGTGTCTGAA |
| *wcaE-*DS-F | TCGCTGACAAAGCGTAGGTT |
| *wcaE-*DS-R | GCATAATCACTGTCGCGTTTC |
| **Primers for promoter** | |
| J23104-F | TTGACAGCTAGCTCAGTCCTAGGTATTGTGCTAGCggaattgtgagcggataacaa |
| J23104-R | GCTAGCACAATACCTAGGACTGAGCTAGCTGTCAAatttcgattatgcggccgtg |
| J23118-F | TTGACGGCTAGCTCAGTCCTAGGTATTGTGCTAGCggaattgtgagcggataacaa |
| J23118-R | GCTAGCACAATACCTAGGACTGAGCTAGCCGTCAAatttcgcgggatcgagat |
| J23107-F | TTTACGGCTAGCTCAGCCCTAGGTATTATGCTAGCggaattgtgagcggata |
| J23107-R | GCTAGCATAATACCTAGGGCTGAGCTAGCCGTAAAatttcgattatgcggc |
| J23111-F | TTGACGGCTAGCTCAGTCCTAGGTATAGTGCTAGCggaattgtgagcggataac |
| J23111-R | GCTAGCACTATACCTAGGACTGAGCTAGCCGTCAAatttcctaatgcaggag |
| **Primers for RBS** | |
| BBa-B0064-F | TCTAGAGAAAGAGGGGAAAtatacatATGAATGAAATTATACTAAT |
| BBa-B0064-R | TTTCCCCTCTTTCTCTAGAgcttactcacggctaacacgacg |
| pET-BBa-B0030-F | TCTAGAGATTAAAGAGGAGAAAtatactaATGGCGCAGTCGAAACTCTA |
| pET-BBa-B0030-R | TTTCTCCTCTTTAATCTCTAGAtgcctgatgcgacgttta |
| pET-BBa-B0032-F | TCTAGAGTCACACAGGAAAGtatactaCCATCTTAGTATATTAGTTA |
| pET-BBa-B0032-R | CTTTCCTGTGTGACTCTAGAggtataggaattgttatccgc |
| pAC-BBa-B0030-F | TCTAGAGATTAAAGAGGAGAAAtatacatATGAAATTTCCCGGTAAA |
| pAC-BBa-B0030-R | TTTCTCCTCTTTAATCTCTAGActaagatggggaattgt |
| pAC-BBa-B0032-F | TCTAGAGTCACACAGGAAAGtatactaATGCAAAAATTGTTAAGTC |
| pAC-BBa-B0032-R | CTTTCCTGTGTGACTCTAGAtgcctttaataggtgatcc |
